# Recent Advances in Inflammatory Skin Diseases: Future Research on Viral Reactivations and Vaccine Improvement

**DOI:** 10.3390/jcm13237347

**Published:** 2024-12-02

**Authors:** Giulia Ciccarese, Astrid Herzum, Luigi Pisano, Caterina Foti, Francesco Drago

**Affiliations:** 1 Department of Medical and Surgical Sciences, University of Foggia, 71122 Foggia, Italy; 2 Private Practice of Dermatology and Venereology, 16145 Genoa, Italy; astridherzum@yahoo.it; 3 Section of Dermatology, Health Sciences Department, University of Florence, 50100 Florence, Italy; luigi.pisano88@yahoo.it; 4 Section of Dermatology and Venereology, Department of Precision and Regenerative Medicine and Ionian Area (DiMePRe-J), University of Bari “Aldo Moro”, 70121 Bari, Italy; caterina.foti@uniba.it; 5 Casa di Cura Villa Montallegro, 16145 Genoa, Italy; francescodrago007@gmail.com

In recent decades, the knowledge on the pathogenesis, immune mechanisms, and molecular signaling pathway underlying inflammatory skin diseases has substantially improved, along with the management of such diseases. The use of antibodies neutralizing disease-associated cytokines and small molecules (such as the Janus kinase [JAK] inhibitors) targeting the intracellular pathways of cytokine production and transducing cytokine-mediated signals involved in the inflammatory process, is widely employed both in common chronic cutaneous diseases (psoriasis, atopic dermatitis) [1,2,3,4] and in rare dermatosis, like pyoderma gangrenosum [5]. Monoclonal antibodies may possibly offer therapeutic potential in treating chronic wounds (such as pyoderma gangrenosum, ulcerated necrobiosis lipoidica, hidradenitis suppurativa, and other atypical wounds) recalcitrant to standard therapies. However, the knowledge on their role is currently limited as well as their use as therapeutic options in vulnerable patients, like pregnant and breastfeeding women [6] and patients with infectious or neoplastic comorbidities [7,8].

Aiming to fill these knowledge gaps, the Special Issue “Recent Advances in Inflammatory and Infectious Skin Diseases” of the *Journal of Clinical Medicine* concerns a variety of topics in the field of inflammatory skin diseases describing the clinico-pathological findings of rare cutaneous diseases that occur in conditions of fragility such as pregnancy, postpartum, and hematoproliferative disorders (Table 1; Contributions 1 and 2).

The successful treatment modalities of pyoderma gangrenosum during pregnancy and lactation have been extensively reviewed in addition to the role of monoclonal antibodies in managing chronic wounds from 16 different etiologies (Contribution 3). In addition, the Special Issue explored some specific aspects of atopic dermatitis treatment: the occurrence of adverse events in patients undergoing anti-interleukin (IL)-4 and IL-13 drugs and the alternative therapeutic options (Contribution 4) as well as the role of the caregiver and the rising issue of corticophobia among parents of children with atopic dermatitis (Contribution 5).

The studies included in this Special Issue underscore the importance of the multidisciplinary management of the chronic inflammatory skin diseases. Interdisciplinary and multi-professional team care, involving dermatologists, gynecologists, oncologists, hematologists, ophthalmologists, and other specialists, is crucial to comprehensively manage conditions refractory to standard therapies, fragile patients, and adverse events [9].

However, open questions on the knowledge of the comorbidities sometimes associated with inflammatory skin diseases already exist, and future research should be focused on the infectious complications that may occur with the use of immunosuppressive agents. Indeed, in cutaneous inflammatory diseases, the immune system may be compromised not only by the chronic inflammation involved in the pathogenesis of the diseases but also by the immunosuppressive treatments [10].

For instance, atopic dermatitis is characterized by epidermal barrier defects, decreased antimicrobial peptide expression, and altered innate and adaptive immunity in the setting of T-helper (Th) 2-skewed inflammation, which can increase susceptibility to infections [11]. Indeed, the administration of immunosuppressive therapies in patients with atopic dermatitis and with other skin immune-mediated diseases further increases the risk of acquiring infections and/or the risk of reactivation of latent viruses. The awakening of viruses from latency can cause different cutaneous manifestations, ranging from self-limiting exanthematous diseases, like pityriasis rosea due to the endogenous systemic reactivation of human herpesvirus (HHV)-6 and HHV-7 [12], to more painful and disabling conditions like herpes zoster caused by the reactivation of Varicella Zoster virus (VZV)12 or erythema nodosum and Stevens–Johnson syndrome associated with acute Epstein–Barr virus infection [13].

The systemic reactivation of herpesviruses is a well-known, dreaded complication in immunocompromised patients receiving hematopoietic stem cell transplant (HCT). After an HCT and myeloablative conditioning regimen, HHV-6 reactivates in 30–70% of patients between 2 and 4 weeks, and it could be an important cause of fever, hepatitis, encephalitis or central nervous system dysfunction, bone marrow suppression, acute graft-versus-host disease, pneumonitis, nephrotoxicity, rash, and mortality in a small proportion of patients [14,15,16]. Occasionally, HHV-6 reactivation may also cause delayed engraftment and allograft dysfunction [17,18,19].

Furthermore, EBV and Cytomegalovirus (CMV) reactivation has been associated, mainly under immunosuppression, with lymphoproliferative disorders, malignancies (B-cell lymphomas and nasopharyngeal carcinoma), pneumonia, ulcers, and fever, respectively [20,21,22].

In contrast to HCT patients who are routinely monitored for herpesvirus plasma load to precociously detect viral reactivation and related complications [10], the patients suffering from chronic skin diseases under immunosuppressive treatment have not yet been studied in detail from this point of view.

A recent interesting study screened 206 patients suffering from chronic skin diseases (psoriasis, atopic dermatitis, urticaria, bullous pemphigoid, pyoderma gangrenosum, and others) to investigate whether the subclinical reactivation of EBV and CMV may have clinical relevance. The authors found that patients under immunosuppressive therapy with treatment-refractory disease showed the reactivation of EBV and CMV infection, differently from non-immunocompromised patients [10]. Immune dysfunction in these patients is also demonstrated by the fact that T cells isolated from lesional skin had an increased proliferation (up to 14-fold) with the production of T helper type 1 and type 17 cytokines upon stimulation with viral proteins, providing evidence for possible aggravation of the underlying skin diseases by the viral infections. Moreover, immunocompromised patients with treatment-refractory disease and reactivation of CMV showed improvement of skin lesions via specific antiviral treatment (ganciclovir) [10].

These data emphasize the usefulness of investigating the viral reactivations in cases of therapy-refractory chronic skin diseases. Virologic investigations could be expanded to include not only EBV and CMV, as described in the study by Speth P. et al. [10], but also herpes simplex virus 1 and 2, varicella zoster virus, HHV-6, HHV-7, and HHV-8. Indeed, early the detection of viral reactivation could reduce morbidity and health care costs [10].

Future research should also emphasize the importance of preventing infections and viral reactivations in immunosuppressed dermatologic patients promoting the available vaccinations based on the most updated version of the National Vaccine Prevention Plan [23]. It is noteworthy to mention that a recent survey conducted among Italian oncologists highlights the urgent issues of training oncologists in vaccine-preventable diseases and vaccine awareness and the necessity to increase a network of multidisciplinary collaborations [24], possibly involving, among others, dermato-venereologists.

## Figures and Tables

**Table 1:** Summary of the papers published in the Special Issue “Recent Advances in Inflammatory and Infectious Skin Diseases” of *Journal of Clinical Medicine*.

Contribution Number	Reference	Type of Publication	Number of Authors/Affiliation	Location of Authors’ Affiliation
1	Matasariu D.R. et al. Pyoderma Gangrenosum, a Challenging Postpartum Diagnosis-Case Report and Literature Review. *J. Clin. Med.* **2024** Jun 22;*13*(13):3653. doi:10.3390/jcm13133653.	Review	10/6	Romania
2	Michelerio A. et al. Eosinophilic Dermatosis of Hematologic Malignancy: Emerging Evidence for the Role of Insect Bites-A Retrospective Clinico-Pathological Study of 35 Cases. *J. Clin. Med.* **2024** May 16;*13*(10):2935. doi:10.3390/jcm13102935.	Article	6/5	Italy
3	Manzo Margiotta F. et al. Monoclonal Antibodies in the Management of Inflammation in Wound Healing: An Updated Literature Review. *J. Clin. Med.* **2024** Jul 12;*13*(14):4089. doi:10.3390/jcm13144089.	Review	9/3	Italy
4	Paganini C. et al. Impact of Upadacitinib on Atopic Keratoconjunctivitis Exacerbated by Dupilumab Treatment in Atopic Dermatitis Patients: A Prospective Dermatological and Ophthalmological Clinical Evaluation in Common Clinical Practice. *J. Clin. Med.* **2024** Jun 28;*13*(13):3818. doi:10.3390/jcm13133818.	Article	8/3	Italy
5	Herzum A. et al. Corticophobia among Parents of Children with Atopic Dermatitis: Assessing Major and Minor Risk Factors for High TOPICOP Scores. *J. Clin. Med.* **2023** Oct 27;*12*(21):6813. doi:10.3390/jcm12216813.	Article	5/1	Italy
